# Exploring feedback and feedforward in dental education using a followership model

**DOI:** 10.3389/fdmed.2026.1822297

**Published:** 2026-05-13

**Authors:** Christina Gummesson, Nina Lundegren

**Affiliations:** Faculty of Odontology, Malmö University, Malmö, Sweden

**Keywords:** clinical education, dental education, digital portfolio, feedback, feedforward, followership

## Abstract

**Objectives:**

The study aimed to explore the feedback and feedforward quality in the clinical setting and the usability of a followership model as a framework to analyze and map students' peer feedback.

**Method:**

Feedback and feedforward from 59 fourth-year dental students' clinical portfolios were collected and analyzed using a combination of deductive coding and interpretative content analysis. The analysis was first organized into predefined code categories (specific and actionable, non-specific, confirmatory, description of what was done, and other comments). The comments provided to peers were clustered and analyzed in relation to Kelley's theoretical followership framework.

**Results:**

A total of 3,877 free-text comments were included in the analysis. Regardless of role, most comments were coded as specific and actionable (*n* = 3,012). Confirmatory comments were frequently directed at peers. Comments that were only confirmatory were defined as reflecting dependent, uncritical thinking and passive behavior; this was the most frequent category among peer comments (*n* = 656). Non-specific comments were defined as reflecting dependent, uncritical thinking and active behavior, and this category was rare (*n* = 79). Comments that were confirmatory, specific, and actionable were defined as expressions of independent, critical thinking and active behavior (*n* = 442).

**Conclusion:**

The analysis of feedback and feedforward in relation to followership seems to be a valid conceptual model. We found that peer feedback was often confirmatory and could be improved by incorporating specific and actionable feedforward. The followership model was shown to be a useful tool for structuring and analyzing peer feedback and feedforward, and it will be used as a tool for learning and quality assessment in future longitudinal studies.

## 1 Introduction

The experience of the physical, organizational, and social work environment depends in part on how collegial collaboration functions in a workplace or study environment (both physical and digital). Within the organizational and social work environment, followership/employee engagement has been shown in various contexts to be important for workplace well-being (1–4). Followership is closely related to leadership. In leadership research, there has been a shift from focusing solely on leadership to a broader perspective that includes followership as part of leadership and organizational processes. There are various definitions of followership (5). We define followership as employees' interactions with leaders and within the organization, from a motivation-oriented perspective in which individuals contribute to improvement and help achieve group and organizational goals through constructive influence (6, 7). Previous studies have shown that the organizational and social work environment affects quality in dentistry, including both the well-being of individual dentists, where stress and burnout are common, and the quality of the service provided (8). It is therefore important to incorporate followership and leadership skills into dental education.

Further, universities are responsible for the work environment of both students and employees. The national degree ordinance also makes it clear that students should develop advanced abilities to work collaboratively with others: “demonstrate advanced ability for teamwork and collaboration with all professional groups within dentistry, as well as with other professional groups in health and social care” (Degree Ordinance). It is possible, even early in education, to raise awareness of everyone's participation and responsibility for the social work environment to promote well-being in the educational context. In recent years, international attention has been drawn to the need for more educational initiatives to support students' development of leadership—and especially followership— competencies within undergraduate programs (3, 9, 10). Existing studies also show that current leadership courses have a limited impact and that further development is needed (2).

An important part of the social work environment is how people interact and communicate, including how they provide feedback to one another. Developing students' feedback skills and followership competence may support improvements in the work environment during their studies and in their future professional lives. Within dental education, there are ample opportunities for collaboration and for exchanging oral and written feedback. This feedback and feedforward are typically used just-in-time but are also recorded. Feedforward to oneself, to peers, and from teachers should not be merely a recording activity. Rather, its content is intended to influence behavior, motivate, foster engagement, and promote lifelong learning.

To understand leadership and followership, Kelley developed a model with four quadrants, with the *x*-axis representing active–passive behavior and the *y*-axis representing dependent, uncritical thinking to independent critical thinking (11). The model has the potential to map and support the development of thinking and behavior, including the mapping and development of feedback/feedforward communication to improve followership and the work environment.

In this project, we sought to explore whether the rich body of feedback generated throughout the dental program could be utilized as a resource to analyze students' followership patterns and whether the quality of the comments reflected actionable and specific feedback that supports development needs.

The aim was to explore the feedback and feedforward quality in the clinical setting and to explore the usability of a followership model as a framework for analyzing and mapping students' peer feedback.

## 2 Methods

### 2.1 Context

The 5-year dental program at Malmö University enrolls approximately 90 students each year. From the first semester, students engage in peer review and self-reflection within the theoretical components of education. From year 2 onward, they provide themselves and their peers with frequent feedback during clinical training and regularly receive feedback from both peers and teachers. No guidelines are provided that feedback/feedforward should reflect a particular feedback model or followership model.

Students maintain a digital portfolio for learning and assessment. During clinical training, the portfolio includes digital forms with specific criteria to assess autonomy and the need for supervisor support, along with the option to add a free-text comment for each criterion. At the end of each form, there is a free-text field titled “Tips to think about the next time.” The forms can be completed by the student for self-reflection, by a peer student, and by a teacher.

### 2.2 Data collection

During the 4th year of study, students are placed in four different clinics with different clinical teachers, working within the same student group for 6 months before rotating to a new student group.

We decided that analyzing the diverse data from one cohort of 4th-year students (59 students) would be suitable for this exploratory study. We retrieved a data set containing information on year, course, role, comments, and the location of each comment (either in the free-text field or as a comment on a specific criterion).

### 2.3 Ethics

All data that form part of the assessment are, under Swedish law (“The freedom of the press act,” TF 2 and “The public access to information and Secrecy Act,” OSL), recognized as public documents and can be retrieved from the university. The data set contained no student identifiers and was not classified as confidential.

### 2.4 Analysis

The analysis followed the principles of content analysis and was conducted in three steps. A deductive orientation guided the initial phase, after which the analysis became increasingly interpretative to evaluate the suitability of the theoretical followership framework and to explore role-based patterns in the material.

During the analysis, information about the role and location of the text was concealed to ensure that it did not affect the interpretation.

#### 2.4.1 Step 1: Deductive coding based on predefined categories

A deductive content analysis was conducted by both authors using a set of predefined codes derived from common principles of feedback and feedforward (specific and actionable, non-specific, confirmatory) and other aspects (description of what was done and other comments). These codes were developed during a pilot study using a different data set.

Following the structured approach described by Hsieh and Shannon (12), the material was reviewed and meaning units were coded. Comments were discussed to reach an agreement on their interpretation. This initial step ensured a systematic organization of the large data set. Excerpts belonging to each code within the framework were then summarized to examine their usage. These summaries were organized by participant roles to identify patterns and differences across roles. This step provided an overview of how frequently and in what ways different roles articulated aspects represented within each category, thereby contributing to a nuanced understanding of role-related variations in the data. To illustrate the codes, quotes were selected.

#### 2.4.2 Step 2: Clustering of codes in relation to a theoretical followership framework

The purpose of this step was to explore whether the framework could be used to map peer comments in relation to followership development. The deductively generated codes were clustered according to the theoretical followership framework (11). Based on the framework definitions, we interpreted how feedback could be characterized within each quadrant. The codes from Step 1 were translated into the quadrants of the framework (Table 1). When codes did not align with existing categories, they were reviewed again to determine their relevance for inclusion. To illustrate the followership categories, quotes were selected.

**Table 1:** Description of followership quadrants (derived from the *x*-axis: passive vs. active behavior, and the *y*-axis: uncritical vs. critical thinking) and our translations into feedback content (codes).

Kelley's followership category	Feedback code
Passive behavior and uncritical thinking	Confirmatory
Passive behavior and critical thinking	-
Active behavior and uncritical thinking	Non-specific
Active behavior and critical thinking	Specific + confirmatory

### 2.5 Reflexivity

The authors are well acquainted with the educational context, with one serving as the program director (NL) and the other as an educational developer (CG). We have dual roles, having led the participatory work process to implement the digital portfolio while also conducting explorative research on its use. The influence of our prior knowledge and invested time in the portfolio project provided valuable insights for interpretation, but we also discussed the impact of this involvement, frequently during the analysis process, to ensure awareness of its possible impact on our interpretations.

## 3 Results

During one semester, the 59 students collected 3,983 instances of free-text comments, either as specific comments directly connected to a criterion or as free text under the heading “tips to think about.” A total of 107 duplicates (when the same form was submitted twice) were removed before the qualitative analysis.

Of the remaining 3,877 free-text comments, the initial deductive analysis was conducted based on the predefined codes; examples of comments are presented in Table 2. When exploring feedback in relation to roles, it was found that 1,608 comments were self-directed, 1,310 were directed to peers, and 1,006 free-text comments were provided by teachers to students.

**Table 2:** Comments from the students’ clinical portfolio forms coded using predefined codes.

Codes	Examples of comments
Confirming	“Great job, you were very independent and skilled!”
	“[dental]Impressions. Everything went as it should.”
	“Good assistance. Got help with everything.”
	“Good that you stayed calm and tried to explain things to the patient in a good way.”
	“Good that you noted the underlying reasons for why the problem occurred.”
	“Good impressions despite a small crest.”
Specific and actionable	“Dab with micro applicator with haemostatic fluid when needed if there is heavy bleeding during excavation; it becomes easier to determine whether it is caries-free.”
	“Ask the patient to show the exercises to see whether they know them or not.”
	“Think about ergonomics and lowering the patient so you can work more ergonomically.”
	“Tip for next time: air spray properly so you can determine whether filling material remains or if it is tooth substance.”
	“Remember to use terms the patient can understand.”
Non-specific	“Think about ergonomics!”
	“Try to place the matrix band better.”
	“Have a treatment plan in mind.”
	“Be clearer in your clinical notes.”
	“Consider prognosis and risk.”
Description of what was done	“Had a cancellation, so assisted instead.”
	“Fissure sealing.”
	“Impressions for a splint.”
	“Try-in of removable prosthetics.”
	“Check of crown after cementation.”

Examples for each code are provided.

Independent of role, most comments were coded as specific and actionable. Confirmatory comments were frequently directed to peers (Table 3).

**Table 3:** Deductive analysis of the 3,877 free-text comments.

Deductive codes	Total	Self	Peer	Teacher
	*N*	*N*	*N*	*N*
Confirming	998	228	658	114
Specific and actionable	3,012	1,200	1,003	808
Non-specific	197	88	79	30
Description of what was done	523	278	124	127
Other comments	129	72	24	33

Each comment could be coded into several codes.

The free-text comments were to oneself (*n* = 1,608), to peers (*n* = 1,310), and from teachers (*n* = 1,006).

**Figure 1:**
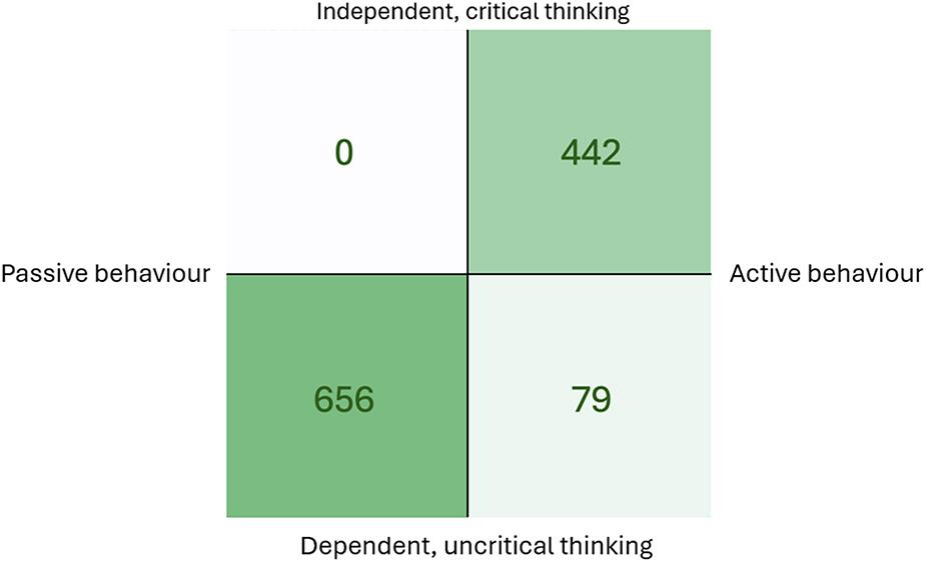
Applying Kelley's framework for followership to peer feedback content. The quadrants represent the dimensions of passive vs. active behavior and dependent, uncritical vs. independent, critical thinking. The lower-left quadrant contains confirmatory comments (*n* = 656). The lower-right quadrant includes non-specific comments (*n* = 79). No comments were classified as passive and critical. The upper-right quadrant contains comments that are confirmatory, specific, and actionable (*n* = 442).

To analyze the content in relation to the followership model, the comments directed to peers were selected. Comments that were only confirmatory were defined as reflecting dependent, uncritical thinking and passive behavior; this was the most frequent category among peer comments (*n* = 656). Non-specific comments were defined as reflecting dependent, uncritical thinking and active behavior, and this category was rare (*n* = 79). Comments that were confirmatory, specific, and actionable were defined as expressions of independent, critical thinking and active behavior (*n* = 442) (Figure 1).

Free-text comments that were confirming, specific, and actionable related to technical skills and performance are as follows:

Bite registration for a complete upper denture, tooth setup with bite rim. Well done. You have taken the reline impression; remember to press up very firmly while asking the patient to bite together so that it seats correctly.

Great job with the impression post today. One tip is to check for undercuts. These will appear as bulges in the impression.

The performance of soft skills, such as communication, was also addressed in various situations, along with suggestions for how these skills could be improved in future instances.

Great job, remember to ask more follow-up questions, especially regarding work situation, social factors, etc.

You have good communication with the patient, well done. However, keep in mind that when working with sharp instruments, you should not “throw” them onto the tray at random, as there is a risk of injury. Also remember that when adjusting a denture with flow material, to remove excess material at the rim, as it will otherwise feel sharp to the patient.

## 4 Discussion

This study explored the quality of feedback and feedforward in the clinical setting and the usability of a followership model as a framework for analyzing and mapping student's peer feedback. We found that the followership model (11) could be used to map the comments. Three of the quadrants matched the codes very well. One of the quadrants was determined not to be applicable to this data set (being critical and passive), as this is undesired and may not be reflected in written feedback or feedforward. We feel that the model has the potential to clarify important aspects of followership development and could serve as a valuable tool for guiding feedback structures, improving communication skills, and promoting a positive work environment.

It is important to assess the quality and content of feedback and feedforward within students' current feedback culture, as this may create a foundation for being good colleagues, both as students and later as practicing dentists. The habit of frequent feedback and feedforward may promote students' confidence and engagement, shape the educational experience, and enhance readiness for work life. In future research, the model will be applied to longitudinal data to explore changes after interventions aimed at improving the quality of feedback and feedforward.

The feedback and feedforward given to peers were often confirmatory and focused on praising students' performance. This may be interpreted as a willingness to encourage and support peers, thereby creating a positive work environment. However, to further promote learning, the inclusion of actionable, constructive comments is important. Several studies have shown that providing feedback benefits not only the receiver but also the giver, improving the student's own performance (13–15).

Most feedback and feedforward were directed to the student themselves, followed by their peers. Based on our deductive analysis, we found that a high proportion of the comments were classified as actionable and specific, thus potentially serving as a scaffold for learning (14, 15). A limitation of this study was that we did not ask the students about their perceptions of the usefulness.

We believe that using Kelley's followership model to analyze feedback could be transferable to other settings. Followership and leadership are closely connected to communication culture, which is important both within education and in future professional life.

### 4.1 Limitations

The feedback and feedforward were extracted in isolation, even though they were recorded in a digital form that included specific criteria and an autonomy scale. This extraction could influence the interpretation of whether a comment was specific or non-specific. We acknowledged this limitation; however, we decided to analyze without the criteria and scale, as students typically review their comments as a list unless they actively reopen the forms. Further, all data were drawn from a single student cohort, limiting the transferability of the results but confirming the transferability of the analysis model.

Another limitation is our dual roles: working on portfolio development and analyzing the content. However, this intertwined working process is important for portfolio development and for the design of learning activities aimed at improving their quality. Throughout the process, we regularly reflected on our dual roles to remain cautious in our interpretation of the findings. The strength, however, is that we know the context well, making interpretations authentic.

A risk of conducting a deductive analysis is overlooking unexpected insights and thereby reducing flexibility. In our study, no free-text comments were left uncoded. A deductive model was found to be suitable for categorizing the large data set of comments. We developed our interpretation of how feedback could be described in the followership model based on our initial codes. However, this interpretation requires further validation.

To explore the analysis model, we considered it important to select a student cohort that was exposed to various challenges during their clinical training, collaborated with multiple peers, and was supervised by different teachers.

## 5 Conclusions

The analysis of feedback and feedforward in relation to followership represents a valid conceptual model. We found that peer feedback was often confirmatory and could be improved by incorporating more specific and actionable feedforward. The followership model proved to be a useful tool for structuring and analyzing peer feedback and feedforward and will be used as a tool for learning and quality assessment in future longitudinal studies.

## Data Availability

The raw data supporting the conclusions of this article will be made available by the authors, without undue reservation.
